# Stabilization Strategies for Fast Walking in Challenging Environments With Incomplete Spinal Cord Injury

**DOI:** 10.3389/fresc.2021.709420

**Published:** 2021-08-19

**Authors:** Tara Cornwell, Jane Woodward, Wendy Ochs, Keith E. Gordon

**Affiliations:** ^1^Department of Physical Therapy and Human Movement Sciences, Northwestern University, Chicago, IL, United States; ^2^Shirley Ryan AbilityLab, Chicago, IL, United States; ^3^Research Service, Edward Hines Jr. VA Hospital, Hines, IL, United States

**Keywords:** gait, walking speed, stability, spinal cord injury, maneuvers

## Abstract

Gait rehabilitation following incomplete spinal cord injury (iSCI) often aims to enhance speed and stability. Concurrently increasing both may be difficult though as certain stabilization strategies will be compromised at faster speeds. To evaluate the interaction between speed and lateral stability, we examined individuals with (*n* = 12) and without (*n* = 12) iSCI as they performed straight walking and lateral maneuvers at Preferred and Fast treadmill speeds. To better detect the effects of speed on stability, we challenged lateral stability with a movement amplification force field. The Amplification field, created by a cable-driven robot, applied lateral forces to the pelvis that were proportional to the real-time lateral center of mass (COM) velocity. While we expected individuals to maintain stability during straight walking at the Fast speed in normal conditions, we hypothesized that both groups would be less stable in the Amplification field at the Fast speed compared to the Preferred. However, we found no effects of speed or the interaction between speed and field on straight-walking stability [Lyapunov exponent or lateral margin of stability (MOS)]. Across all trials at the Fast speed compared to the Preferred, there was greater step width variability (*p* = 0.031) and a stronger correlation between lateral COM state at midstance and the subsequent lateral foot placement. These observations suggest that increased stepping variability at faster speeds may be beneficial for COM control. We hypothesized that during lateral maneuvers in the Amplification field, MOS on the Initiation and Termination steps would be smaller at the Fast speed than at the Preferred. We found no effect of speed on the Initiation step MOS within either field (*p* > 0.350) or group (*p* > 0.200). The Termination step MOS decreased at the Fast speed within the group without iSCI (*p* < 0.001), indicating a trade-off between lateral stability and forward walking speed. Unexpectedly, participants took more steps and time to complete maneuvers at the Fast treadmill speed in the Amplification field. This strategy prioritizing stability over speed was especially evident in the group with iSCI. Overall, individuals with iSCI were able to maintain lateral stability when walking fast in balance-challenging conditions but may have employed more cautious maneuver strategies.

## Introduction

A major goal of gait rehabilitation following incomplete spinal cord injury (iSCI) is to improve the ability of a patient to participate in a variety of walking activities (1). Training interventions often aim to simultaneously enhance both walking speed and stability, i.e., the ability to reestablish an intended walking trajectory following a perturbation (2). However, achieving concurrent increases in speed and stability may be particularly challenging in a population with iSCI and known balance deficits (3) because common strategies believed to aid in the maintenance of stability will be directly compromised as gait speed increases.

Although ambulatory individuals with iSCI typically walk slowly (4), it is not clear if slower walking speeds are a necessary compensatory strategy that is used to mitigate balance deficits or a result of injury that reduces stability. Slower walking speeds have several potential advantages for maintaining stability including the following: (1) increasing stride duration (5), allowing for greater time to make within-step error corrections, (2) increasing double support time when the base of support is the greatest (5), and (3) minimizing the angular momentum of the limbs and center of mass (COM), which reduces the forces and coordination required to control movements (6). However, some characteristics of slow walking may be detrimental to stability. Slow walking speeds are associated with increases in lateral COM excursion (7), which may increase the requirements of the nervous system to maintain lateral stability, a degree of freedom requiring active control (8, 9). Walking slowly is also associated with greater variability in stride time (10, 11), which has been identified as a risk factor for falling in older adults (12, 13). Past research has examined the relationship between walking speed and stability in populations without neurological injury. Some found that forward walking speed has no direct effect on stability (14), while others found that walking slowly is more “stable” but also more variable (10, 11). Collectively, these prior mixed findings in other populations and the additional challenges to balance and coordination for those with iSCI make it unclear how speed and stability relate in this population. Therefore, our purpose was to examine the relationship between walking speed and the control of lateral stability in ambulatory individuals with iSCI. A better understanding of the speed-stability relationship in people with iSCI could inform clinical decision-making and the design of safer, more effective gait rehabilitation interventions.

To evaluate the interaction between walking speed and control of lateral stability in this study, individuals with and without iSCI walked at Preferred and Fast treadmill speeds during two conditions intended to alter the requirement to maintain lateral stability. We challenged lateral stability because normal treadmill walking may not provide sufficient stimuli to detect differences in gait stability. First, a cable-driven robot applied a continuous “movement amplification” force field to the pelvis of each participant during select trials. The forces were proportional to and in the same direction as the real-time lateral velocity of the participant (15). This force field is designed to increase the lateral momentum of the COM during walking and, in populations without neurological injury, has been found to result in reduced local stability of lateral COM velocity, as well as increased lateral margin of stability (MOS), mean step width, and step width variability during treadmill walking (16). The Amplification field was expected to aid in the detection of potential differences in lateral stability and the associated stabilization strategies when walking at different speeds.

As a second challenge to stability, participants also performed a series of lateral “lane-change” walking maneuvers. Compared to straight walking, maneuvers are less locally stable (17) and require a complex generation of mediolateral impulses and compensatory actions (18), so including walking maneuvers was intended to help detect the effects of speed on stability in this study. In people with iSCI, we previously found that the lateral MOS during the initiation step of a maneuver decreases as maneuver speed increases (19). While this previous research did not find an effect of speed on the lateral MOS during the termination steps of a maneuver (19), it is possible that differences might be detectable in more challenging environments. For example, compared to maneuvers with no external forces applied, individuals with iSCI had a smaller lateral MOS during the termination step in an Amplification field (20), indicating a greater challenge to arrest their lateral movement. Therefore, we anticipated that our capacity to evaluate the effect of walking speed on the control of lateral stability in a population with iSCI would be enhanced by utilizing an Amplification force field and by observing a combination of both straight-ahead walking and lateral walking maneuvers.

To test the effect of gait speed on lateral stability during straight walking, we assessed stability using a continuous metric (short-term Lyapunov exponent) and a discrete metric (MOS). Following any small deviations in the kinematic trajectory during walking, the Lyapunov exponent quantifies how the system returns to the mean (21, 22). This measure of local dynamic stability will increase in magnitude as the system becomes less stable. In contrast, lateral MOS is an instantaneous measure based on the inverted pendulum model of walking that quantifies a safety-factor distance between the COM state and the lateral base of support (23). The interpretation of MOS is complex, but during the current steady-state walking task, we expected increases in MOS to be adopted to maintain stability by increasing the safety-factor distance. Thus, a larger MOS could be an adaptive response to the greater challenge to control lateral COM dynamics when walking at increased speeds. We anticipated that individuals with and without iSCI would be able to maintain walking stability at a Fast speed during normal conditions, but the effects of speed would become clear when walking in the Amplification field. Specifically, we hypothesized that both groups would be less stable (greater Lyapunov exponent and lateral MOS) when walking in the Amplification field at the Fast speed compared to the Preferred. In addition, to better understand the strategies contributing to gait stability, we examined step-to-step mediolateral foot placement and its correlation to lateral COM state (position and velocity) during the swing phase (24). A stronger correlation between COM dynamics and the following lateral foot placement may indicate step-to-step adjustments to maintain stability. We expected a stronger correlation between lateral COM state and the subsequent foot placement in the Amplification field (16) and at the Fast speed (25) as the requirements to control lateral COM may increase in both conditions.

To evaluate the effect of forward walking speed on lateral stability during walking maneuvers, we examined minimum lateral MOS at two critical points during the maneuver: Initiation and Termination steps. The MOS was used to evaluate stability because it can capture stability during a singular step of non-steady-state locomotion. During maneuvers, a smaller lateral MOS indicates a reduced mechanical resistance to lateral motion. As previously observed in normal conditions (19), we anticipated that the Initiation step MOS would be smaller at the Fast speed compared to the Preferred, emphasizing the benefits of stability-maneuverability trade-off to facilitate rapid, lateral movement (26). Conversely, we anticipated that participants would not alter MOS on the Termination step, during which the consequences of a smaller MOS may result in an unsuccessful arrest of the maneuver. However, we expected the Amplification field to exaggerate changes in MOS during these steps. Thus, we hypothesized that in the Amplification field, lateral MOS would be smaller at the Fast speed compared to the Preferred on both the Initiation step (due to the Amplification field assisting with the maneuver onset) and the Termination step (due to an inhibited ability to control the increased lateral momentum). To understand the effects of speed on stepping strategy and maneuver performance, we also evaluated changes in step width during the Initiation and Termination steps, as well as the time required to accomplish each maneuver.

## Materials and Methods

### Participants

A total of 27 individuals provided written informed consent to participate. The experimental protocol was approved by both the Edward Hines Jr. VA Hospital and Northwestern University Institutional Review Boards. Fifteen adults with a motor iSCI were recruited from a volunteer spinal cord injury research registry and participated in the study. Of these participants, 12 completed the full testing protocol (three participants with iSCI withdrew because they were unable to complete the protocol without external assistance). Twelve gender- and age-matched (± 5 years) adults without iSCI also participated in this study. All participants were able to walk continuously for 10 min without undue fatigue or health risks. Participants with iSCI also met the following criteria: spinal cord injury between C1-T10 with an American Spinal Cord Injury Association (ASIA) Impairment Scale (AIS) classification of C or D, more than 1-year post-injury, able to ambulate 10 m without assistive devices or orthotics that cross the knee, passive range of motion of the legs within the functional limits of ambulation (i.e., ankle dorsiflexion to neutral, knee flexion of 0–120 degrees, and hip flexion to 90 degrees and extension to 10 degrees). Additional exclusion criteria for participants with and without iSCI included the following: existing orthopedic injury or degenerative condition (outside the diagnosed spinal cord injury) that directly affects ambulation, concomitant central or peripheral neurological injury (e.g., traumatic head injury or peripheral nerve damage in lower limbs), and the use of medications that might affect proprioception or balance (e.g., benzodiazepines, neuroleptics, or opioids).

### Experimental Setup

Participants performed all walking trials on a large treadmill, belt size of 2.6 × 1.4 m, long × wide, respectively (Tuff Tread, Willis, TX). For safety, participants wore a trunk harness attached to passive overhead support that did not provide bodyweight support (Aretech, Ashburn, VA). A 12-camera motion capture system (Qualisys, Gothenburg, Sweden) collected 3D kinematic data from 13 reflective or active LED markers at 100 or 200 Hz, respectively. Markers were placed on the pelvis and feet at the following locations: S2 vertebrae and bilaterally on each sacroiliac joint, the greater trochanter, the lateral malleolus, the calcaneus, and the 2nd and 5th metatarsals. Participants also had four force-sensing resistors (FSRs) fixed to the bottom of their shoes (Delsys Inc., Natick, MA). The FSRs were aligned underneath the heel and the lateral ball of the foot to identify real-time footstep events, i.e., the heel-strike (HS) and the toe-off (TO), with a sampling frequency of 1 kHz. The FSRs triggered visual feedback to cue participants to initiate maneuvers at a specific time in the gait cycle.

To challenge frontal plane stability during select walking trials, a lateral force field was created by the Agility Trainer, a cable-driven robotic device (15). The Agility Trainer consists of two independent series elastic actuators, each of which is powered by a linear motor in series with an extension spring. Linear motors transmit forces *via* a system of cables, pulleys, and trolleys to the lateral aspects of a snug pelvis harness worn by the participant. The cable configuration allows for the movements of the participant in the mediolateral and fore-aft directions. The linear motors are controlled by a cRIO-9074 FPGA with LabVIEW Real-Time software (National Instruments, Austin, TX) to produce bilateral forces that are proportional to the real-time lateral COM velocity of the participants. A closed-loop control scheme uses feedback from optical encoders within the series elastic actuators, which estimate the real-time lateral COM velocity of the participants and directly measure spring extension, and the load cells attached bilaterally at the pelvis, which measure applied forces. The system has a delay of ~30 ms between the input velocity and the output force (15).

### Protocol

First, a licensed physical therapist performed clinical outcome measures to assess the strength and walking function of all participants with iSCI. Assessments of participants with iSCI included: lower extremity motor score (LEMS) from the ASIA Impairment Scale (AIS) (27, 28), Walking Index for Spinal Cord Injury (WISCI II) (29, 30), timed up and go (TUG) test (31), and 10 Meter Walk Test (10MWT) (31–33) at both self-selected and fast speeds. Assessment of participants without iSCI included the TUG and 10MWT at both self-selected and fast speeds.

Next, we identified the Preferred and Fast treadmill walking speeds of the participants. Preferred treadmill speed was confirmed by the participants following a staircase method of small increases and decreases in the walking speed, similar to previously outlined methods (34). The “Fast” treadmill speed of the individual was calculated based on the ratio of overground walking speeds recorded during the 10MWTs. Specifically, each Fast treadmill speed was calculated by dividing the self-selected 10MWT speed of the participants by their fast 10MWT speed, then multiplying this factor by the Preferred treadmill speed of the participants. For safety, the Fast speed was adjusted if the participants were unable to maintain the prescribed speed. Participants were then given 2 min to accommodate to treadmill walking at each speed: Preferred and Fast.

Each participant then performed six trials of treadmill walking. The order of these trials was randomized for each participant. Participants walked on the treadmill at two speeds: (1) Preferred and (2) Fast. These speed conditions were repeated for three lateral force field conditions: (1) Null, no applied forces, (2) Amplification, negative viscous field, and (3) Damping, positive viscous field. In the Amplification and Damping fields, participants experienced forces proportional in magnitude and the same or opposite direction as the real-time lateral COM velocity, respectively. The gains of the Amplification and Damping force fields, which set the relationship between the applied force and lateral COM velocity, were −50 and +50 Ns/m, respectively. For safety, the maximum applied force was restricted to 90 N. During each trial, participants performed two sequential tasks in the following order: (1) straight walking for 200 steps and (2) 12 lateral maneuvers. To narrow the scope of the current results, the data from trials performed in the Damping field are not included in the current analysis.

For the entirety of each trial, participants received visual feedback of their COM location (Supplementary Video 1). A custom LabVIEW program used the real-time position of the S2 marker to estimate the lateral COM position of the participants (35) and projected a feedback line in the fore-aft direction on the treadmill. During straight walking, participants were instructed to do their best to maintain their lateral COM feedback line inside a narrow target lane (0.25 m wide), which was also projected onto the treadmill.

After completing the 200 steps of straight walking, the location of the target lane (0.25 m wide) was systematically relocated to the left or the right side of the treadmill to prompt participants to perform 12 side-to-side walking maneuvers (Supplementary Video 1). The inner distance between the two lanes during maneuvers was 0.05 m. Visual changes in the location of the target lane were accompanied by an audio cue. Target lane changes were triggered when the following criteria were satisfied: COM of the participants, estimated as the location of the S2 marker, entered laterally into the new lane and the required number of steps was completed. The number of steps required in the target lane ranged from 4 to 9 steps, ending on a step contralateral to the maneuver direction. The number of steps was randomized for each maneuver so participants could not predict when the lane change would occur. To limit within- and across-participants variations in the initial conditions of each maneuver, the timing of the target lane changes occurred instantaneously at HS of the foot contralateral to the maneuver direction.

### Kinematic Analysis

Kinematic data were processed with Visual3D (C-Motion, Germantown, MD) and custom MATLAB (Mathworks, Natick, MA) scripts. Marker data were low-pass filtered (fourth-order Butterworth, 6 Hz cut-off frequency) and gap-filled (third-order polynomial with a maximum gap of 10 frames) in Visual 3D. While FSRs were used to detect real-time gait events during the experiment, HS and TO events were identified in post-processing based on the vertical positions of the calcaneus and 2nd metatarsal markers, respectively, and confirmed by visual inspection. COM position and velocity were calculated in Visual3D with the default model of the pelvis and extra tracking markers.

#### Straight-Walking Kinematics

We focused on gait stability metrics related to lateral COM control because a previous study suggests that walking requires active control to maintain frontal plane stability (8, 9). Only the last 100 of the 200 steps of straight walking from each trial were evaluated to reduce bias from the previous trial. To examine the effects of speed and field on COM stability, we calculated the short-term Lyapunov exponent, minimum lateral MOS, and peak lateral COM excursion.

As opposed to metrics that assume purely periodic motion, the non-linear analysis of the Lyapunov exponent quantifies the average logarithmic rate of divergence of a system after small kinematic deviations (21, 22). This metric was calculated from lateral COM velocity and has demonstrated construct and predictive validity in prior simulation and walking studies (36). To convert the time-series data collected [*x(t)*] to the *d*_*E*_-dimensional state-space representation required for this analysis [*X(t)*], we used a time delay (*T*) of 10 samples and an embedding dimension (*d*_*E*_) of 5 (Equation 1) (21).


(1) $$\begin{array}{r} X\left(t\right)=\left[x\left(t\right),x\left(t+T\right),\ldots \;,\;x\left(t+\left(d_{E}-1\right)T\right)\right] \end{array}$$

Then, the log(divergence) curve was calculated from the distances between nearest neighbors, and the short-term Lyapunov exponent was quantified as the slope between 0 and 0.5 strides (37). Higher values indicate a more unstable system with a greater divergence of neighboring trajectories.

Lateral MOS was calculated as the lateral distance between the extrapolated COM (XCOM) position, which is the first term in Equation 2 below, and the base of support (BOS) (23).


(2) $$\begin{array}{r} MOS=\left(COM_{lat}+\frac{{\dot{COM}}_{lat}}{\sqrt{\frac{g}{l}}}\right)-\;BOS \end{array}$$

*COM*_*lat*_ and *CȮM*_*lat*_ consist of the lateral components of COM position and velocity, respectively, *g* is the gravitational constant, and *l* is leg length, estimated by the vertical greater trochanter position. Methods to calculate the minimum lateral MOS have been described in prior studies (26), and in this study, the boundaries of the BOS were defined as the lateral positions of the 5th metatarsal markers during the stance phase. Peak lateral COM excursion was calculated as the maximum lateral distance traveled by the COM between HS events. To examine the effects of speed on stepping and probe potential stabilization strategies, we also evaluated step width and its variability. Step width was calculated as the mediolateral distance between the 5th metatarsal markers at midstance. The SD was calculated to evaluate step-to-step variability, which has been used in prior studies to provide insight into factors that influence stability and potential control strategies.

Finally, to assess the step-to-step maintenance of COM stability, we calculated the correlation between lateral COM state (both position and velocity) at midstance and subsequent lateral foot placement. A previous study has shown that lateral COM state at midstance can predict >80% of the variance in the following lateral foot placement (24). Midstance was defined as the time point halfway between the HS and TO events for every single-leg stance phase. We performed separate linear regression analyses (Equation 3) to predict lateral foot placement from COM state at midstance for each participant during each trial.


(3) $$\begin{array}{r} FP=\;\beta_{0}+\beta_{1}\;*\;COM_{MS}+\beta_{2}\;*\;{\dot{COM}}_{MS} \end{array}$$

*FP* is the mediolateral distance between the lateral malleolus position at HS and the contralateral lateral malleolus position at midstance. β_0_ is the model intercept and other β_*n*_ values are the coefficients of the independent variables. The *COM*_*MS*_ (the lateral COM position relative to the lateral malleolus position on the stance foot at midstance) and *CȮM*_*MS*_ (the lateral COM velocity at midstance) were used to predict FP. Each model included the last 50 steps of straight walking, which was selected based on a recent study that found model convergence to a steady state within 50 steps (25). We recorded the model fit (*R*^2^), which represents how much of the variance in the lateral foot placement could be explained by the COM state at midstance, i.e., a greater *R*^2^ indicates that the model is a good fit and suggests lateral stepping was more coordinated with COM motion.

Other calculated metrics include peak lateral COM velocity per step, step length, step time, and the SDs of the latter two metrics. These additional metrics are reported in Supplementary Table 1 for descriptive purposes but will not be included in the current discussion.

#### Maneuver Kinematics

**Figure 1:**
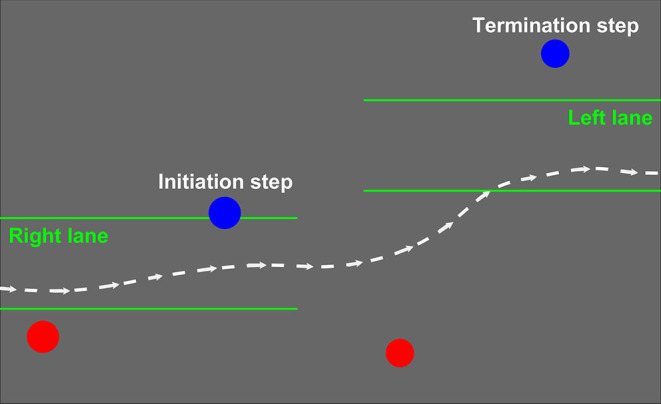
A labeled representative maneuver from the right (bottom) to the left (top) lane. The blue Initiation and Termination steps are the first and last steps of the maneuver ipsilateral to the lane-change direction. The dashed white line represents lateral center of mass (COM) position over time and the green lines represent the lanes projected, one at a time, onto the treadmill surface.

The following metrics were averaged across the maneuvers during each trial: Initiation and Termination step MOS and step width, as well as maneuver time. The Initiation step (Figure 1) was the last step ipsilateral to the maneuver direction immediately prior to the lateral movement of the COM out of the current lane. The Termination step (Figure 1) was the first step ipsilateral to the maneuver direction immediately after the COM entered the new target lane. The Termination step was defined by the first midstance instead of HS because COM can laterally shift during the stance phase between HS and midstance. Since the number of steps to maneuver was not constricted to these four steps, additional steps between the Initiation and Termination step pairs were permitted but not analyzed.

Minimum lateral MOS values for the Initiation and Termination steps were calculated using the same methods outlined above for straight walking. Step width was calculated at midstance as the mediolateral distance between 5th metatarsal markers of the Initiation and Termination steps and the contralateral steps preceding these defined steps.

To evaluate maneuver performance, we also calculated maneuver time. Maneuver time was defined as the time it took for the COM to successfully enter the new target lane after successfully exiting the previous lane.

Since our goal is to understand side-step maneuvers, any maneuvers that were executed with crossover steps were removed from the analysis. A crossover step occurred if the calcaneus marker of the contralateral Termination step was positioned on the ipsilateral side to the maneuver direction compared to the calcaneus marker of the other foot at its prior HS. Of the 576 maneuvers made per group across the four trials, 37 (6.4%) from the iSCI data and 49 (8.5%) from the Non-iSCI data were crossover steps and removed from the analysis.

### Statistical Analysis

#### Straight-Walking Statistical Analysis

To investigate the differences between the two speeds and two fields, separate linear mixed-effects models were generated for each of the gait-related metrics with data averaged per trial per participant (SPSS, IBM, Armonk, NY). Fixed effects included Speed (Preferred and Fast), Field (Null and Amplification), and Group (iSCI and Non-iSCI), as well as the interactions between Speed and Field and between Speed and Group. Random intercepts allowed participants to deviate from the main intercept. If there was a significant main effect, a pairwise comparison was made to determine the significant difference within the pair. If there was a significant interaction between Speed and Field or Speed and Group, separate pairwise comparisons were made to evaluate the significant pair (Preferred vs. Fast) within each Field or Group, respectively. Significance was set at α = 0.05 for all tests.

To quantify the correlation between COM state and subsequent foot placement, we performed linear regression analyses (Equation 3) to predict lateral foot placement from lateral COM position and velocity at midstance. Then, we reported the *R*^2^ value of the model for each participant during each trial. However, there are some notable limitations to this method of analysis. One such limitation is that linear regression models assume every data point is independent, but in this case, every data point in a given model is taken from a single participant. In addition, it is assumed that the true relationship between lateral COM state and subsequent foot placement is linear, but it may be more complex or differ across people, especially those with neurological injury.

#### Maneuver Statistical Analysis

To investigate how maneuver strategies differed across the four conditions, we generated linear mixed-effects models for MOS and step width. Fixed effects included Step (Baseline, which was evaluated from straight walking, Initiation, and Termination), and the interactions between Step, Field, and Speed, as well as between Step, Group, and Speed. For the three-way interactions, trials performed at Preferred and Fast speeds were compared within each field (Null and Amplification) and Group (iSCI and Non-iSCI) separately for the two maneuver steps. Random intercepts allowed participants to deviate from the main intercept. If there was a significant effect of Step, Bonferroni-corrected pairwise comparisons were made to determine the significant pair(s) (Initiation vs. Baseline, Termination vs. Baseline, and Initiation vs. Termination). If there was a significant effect from any of the three-way interactions, pairwise comparisons (Preferred vs. Fast) were made to determine the significant pair(s) within the Initiation and/or Termination steps.

To compare maneuver performance across the four trials, we evaluated maneuver time. Due to the non-normality of the data, we performed a non-parametric Friedman test to compare the maneuver time across trials. If this yielded a significant result, we performed Dunn–Bonferroni *post-hoc* tests to compare Preferred vs. Fast speeds within each field. Significance was set at α = 0.05 for all statistical tests.

## Results

### Participant Demographics

Twenty-four total individuals completed the study protocol. The 12 participants with iSCI were 52.9 ± 12.6 years old, 76.9 ± 11.1 kg in weight, and 11 men/1 woman. Participants were 5 to 39 years post-injury and all classified as AIS D. The cause of spinal cord injury was traumatic injury (e.g., motor vehicle accident or gunshot wounds) for 11 participants and bleeding in and around the spinal cord for 1 participant. Half of the participants with iSCI self-reported falling in the past year with five reporting two or more falls during this period. The clinical outcome measures (Table 1) suggest that this cohort was relatively high functioning and able to ambulate independently in the community (38) without devices or physical assistance (39). The 12 participants without iSCI were 53.1 ± 14.8 years old, 82.6 ± 15.3 kg in weight, and 11 men/1 woman. Preferred and Fast treadmill speeds were 0.55 ± 0.22 and 0.74 ± 0.29 m/s, respectively, for participants with iSCI and 1.03 ± 0.22 and 1.49 ± 0.35 m/s, respectively, for participants without iSCI. The multiplying factors used to calculate the Fast speeds were 1.39 ± 0.28 and 1.48 ± 0.15 for participants with and without iSCI, respectively. Although not included in this report, separate linear mixed-effects models of step length and step time confirmed a significant effect of speed (*p* < 0.001 for both metrics), confirming that participants took longer, faster steps at the Fast speed compared to the Preferred.

**Table 1:** Demographic and clinical information for study participants.

**Sex**	**Age** **(years)**	**Pref. speed** **(m/s)**	**Fast speed** **(m/s)**	**SS** **10MWT** **(m/s)**	**Fast** **10MWT** **(m/s)**	**TUG** **(s)**	**SCI level**	**WISCI** **II**	**Total** **LEM** **Score**
**iSCI participants**									
M	54	0.8	1.0	1.3	1.7	9.0	C4-C5	20	50
M	56	0.4	0.6	0.8	1.2	29.5	C5-C7	20	49
M	62	0.5	0.7	1.2	1.6	10.2	C3-C5	18	44
M	54	0.5	0.7	0.9	1.3	14.3	C4-C7	20	50
M	68	0.5	0.7	1.2	1.4	8.2	C3-C7	20	49
M	34	0.4	0.5	0.8	1.1	13.6	T8	18	35
M	57	1.0	1.4	1.5	2.1	8.1	C6-C7	20	50
M	64	0.6	0.9	0.8	1.1	16.1	C3-C4	20	48
M	57	0.2	0.4	0.3	0.7	32.0	C6-C7	20	39
M	36	0.8	1.0	1.6	1.9	7.9	C7	20	50
F	30	0.5	0.6	0.9	1.0	15.7	T7-T9	20	48
M	63	0.4	0.5	0.6	0.7	19.9	C6	20	38
Mean ± SD	52.9 ± 12.6	0.5 ± 0.2	0.7 ± 0.3	1.0 ± 0.4	1.3 ± 0.5	15.4 ± 8.1	Median (Q1-Q3)	20 (20)	48.5 (41.5–50)
**Non-iSCI participants**									
M	50	1.2	1.7	1.5	2.2	8.0	
M	56	1.1	1.5	1.6	2.1	6.4	
M	64	0.8	1.2	1.1	1.5	10.4	
M	55	1.2	1.6	1.4	1.9	9.5	
M	71	1.0	1.5	1.2	2.0	6.9	
M	30	1.2	2.1	1.4	2.5	8.6	
M	56	1.3	1.9	1.5	2.2	6.7	
M	67	0.7	1.0	1.1	1.8	8.1	
M	62	0.9	1.3	1.3	1.7	8.8	
M	35	0.6	0.9	1.1	1.7	11.0	
F	27	1.2	1.7	1.4	2.1	7.4	
M	64	1.1	1.4	1.3	1.7	10.0	
Mean ± SD	53.1 ± 14.8	1.0 ± 0.2	1.5 ± 0.3	1.3 ± 0.2	2.0 ± 0.3	8.5 ± 1.5	

### Straight Walking

#### Lateral COM Control and Stability Metrics

**Figure 2:**
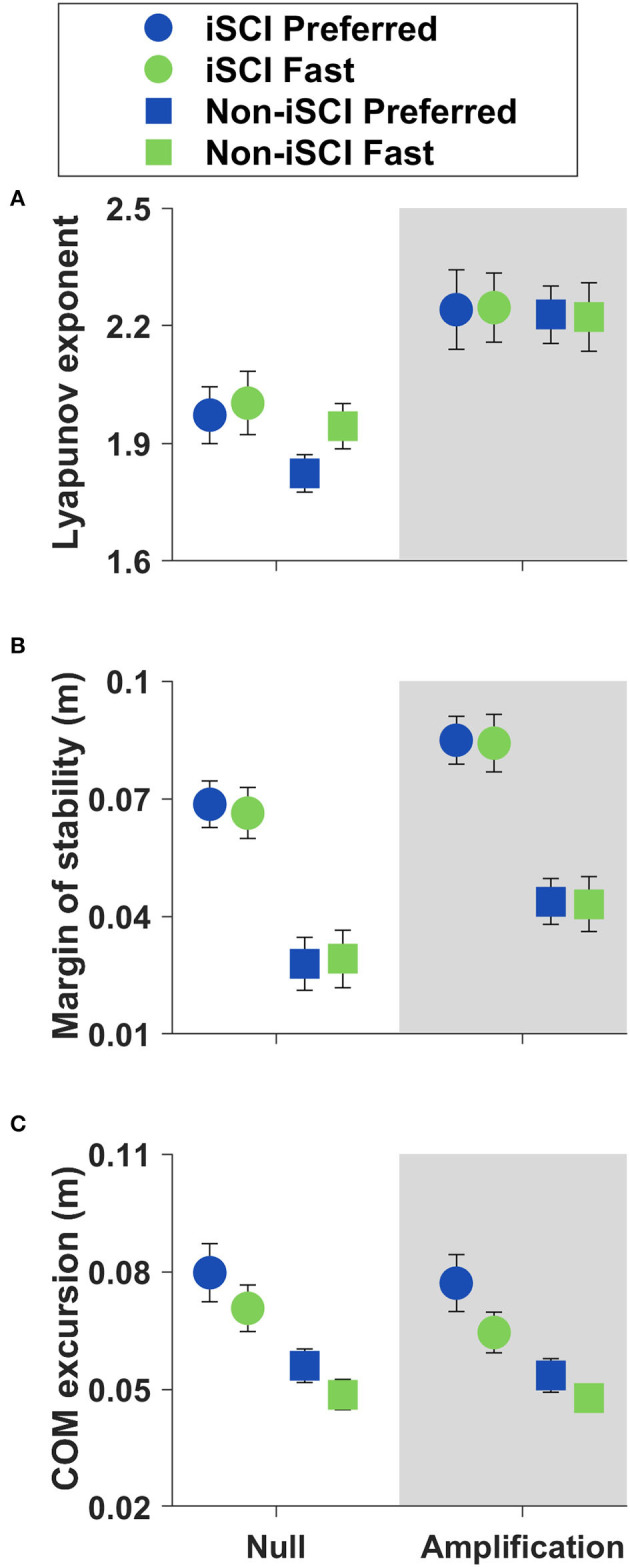
Means ± SE of COM control metrics during straight walking. **(A)** The short-term Lyapunov exponent did not change with speed (*p* = 0.240). **(B)** The minimum lateral margin of stability (MOS) did not change with speed (*p* = 0.664). **(C)** Lateral COM excursion decreased at the Fast speed (*p* < 0.001).

We examined the following metrics that inform us about lateral COM control: local dynamic stability as short-term Lyapunov exponent, minimum lateral MOS, and peak lateral COM excursion (Figure 2 and Table 2). Lyapunov exponent and MOS did not significantly change with speed (*p* = 0.240 and *p* = 0.664, respectively). Lateral COM excursion decreased at the Fast speed compared to Preferred (*p* < 0.001). Lyapunov exponent and MOS both increased significantly in the Amplification field compared to the Null field (*p* < 0.001 for both metrics). However, there was no interaction effect between speed and field for either Lyapunov exponent (*p* = 0.233) or MOS (*p* = 0.940).

**Table 2:** Results from linear mixed-effects models for all straight-walking metrics.

**Metric**	**Factor**	***P*-value**	**Significant pairs**
Short-term Lyapunov exponent	Speed	0.240	
	**Field**	**<0.001**	**Null** **<** **Amplification**
	Group	0.529	
	Speed*Field	0.233	
	Speed*Group	0.538	
Minimum lateral margin of stability	Speed	0.664	
	**Field**	**<0.001**	**Null** **<** **Amplification**
	**Group**	**<0.001**	**iSCI** **>** **Non-iSCI**
	Speed*Field	0.940	
	Speed*Group	0.520	
Peak lateral COM excursion	**Speed**	**<0.001**	**Preferred** **>** **Fast**
	**Field**	**0.007**	**Null** **>** **Amplification**
	**Group**	**0.008**	**iSCI** **>** **Non-iSCI**
	Speed*Field	0.666	
	Speed*Group	0.061	
Step width	Speed	0.096	
	**Field**	**<0.001**	**Null** **<** **Amplification**
	**Group**	**0.001**	**iSCI** **>** **Non-iSCI**
	Speed*Field	0.563	
	Speed*Group	0.500	
Step width variability	**Speed**	**0.031**	**Preferred** **<** **Fast**
	**Field**	**<0.001**	**Null** **<** **Amplification**
	**Group**	**0.010**	**iSCI** **>** **Non-iSCI**
	Speed*Field	0.408	
	Speed*Group	0.244	

*Bold values highlight statistically significant results*.

#### Stepping Metrics

**Figure 3:**
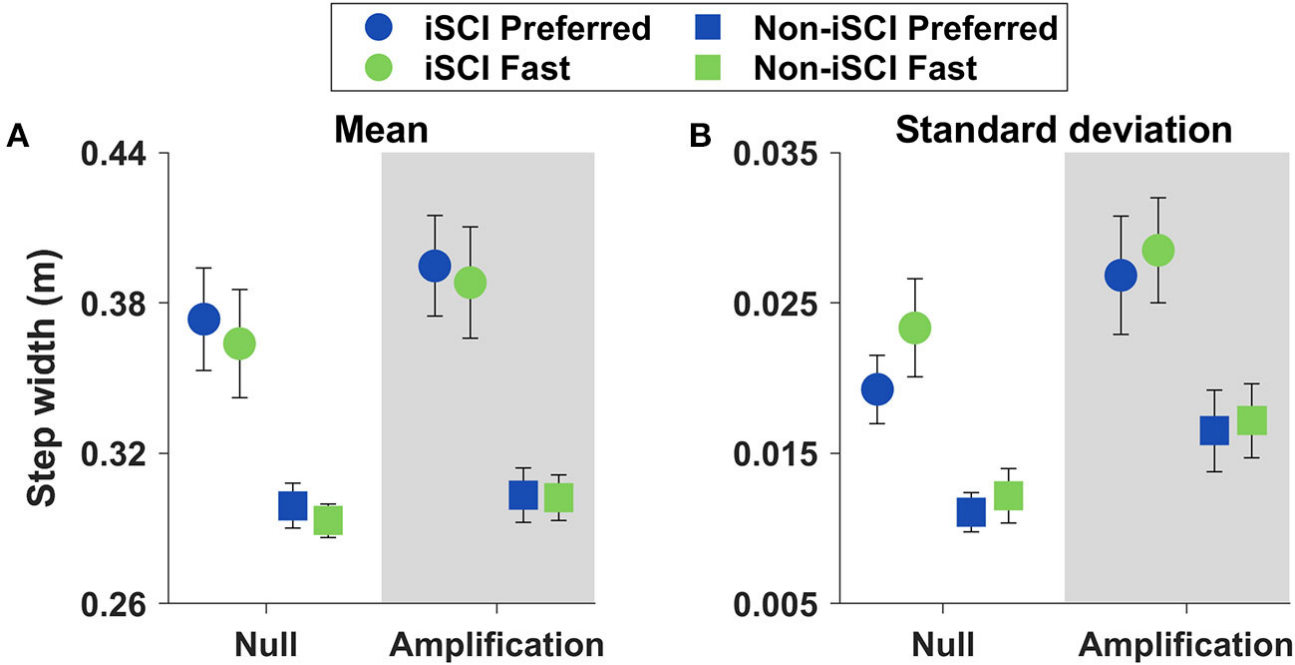
Means ± SE of step width and its variability during straight walking. **(A)** Step width did not change with speed (*p* = 0.096). **(B)** Step width variability increased at the Fast speed (*p* = 0.031).

To examine potential control strategies that contribute to stability, we looked at step width average and variability (Figure 3 and Table 2). Step width was not significantly affected by speed (*p* = 0.096), but it demonstrated greater variability at the Fast speed (*p* = 0.031).

#### Analysis of COM State and Lateral Stepping Correlation

**Figure 4:**
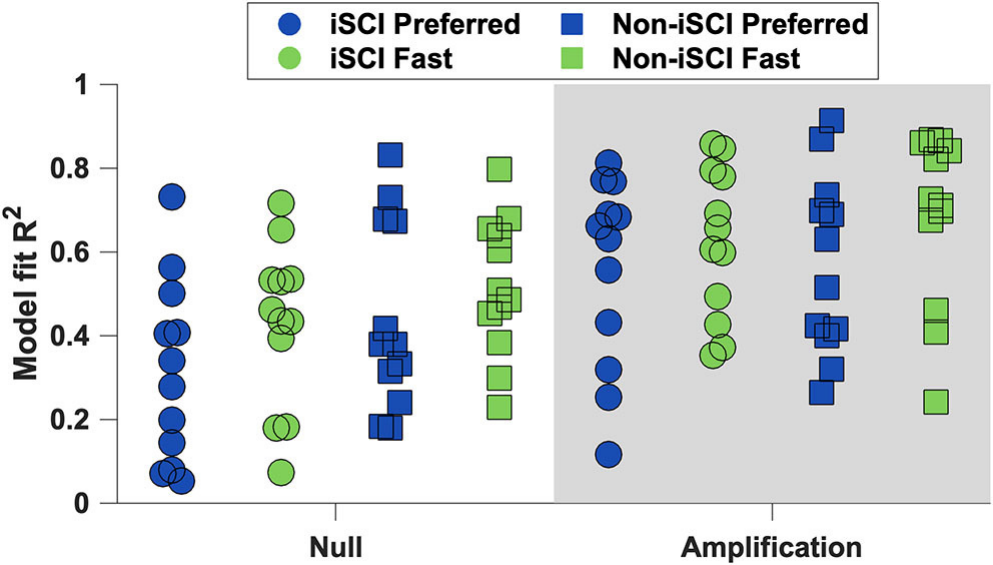
The linear regression models' *R*^2^ values for each participant. The trends suggest that participants had a stronger correlation between lateral COM state and subsequent foot placement during trials at the Fast speed and in the Amplification field.

We quantified the relationship between lateral COM state at midstance and the subsequent lateral foot placement for every participant walking in the four experimental conditions (Figure 4). Each model produced a measure of goodness-of-fit, but these *R*^2^ values cannot be directly compared because every model uses different data. Therefore, we report that the trends suggest a stronger linear correlation between lateral COM state at midstance and subsequent lateral foot placement during trials performed at the Fast speed and in the Amplification field. In addition, trends in *R*^2^ magnitude suggest individuals with iSCI walk with weaker coordination between COM state and foot placement than their age-matched peers without iSCI.

### Walking Maneuvers

#### Maneuver Strategy Metrics

**Figure 5:**
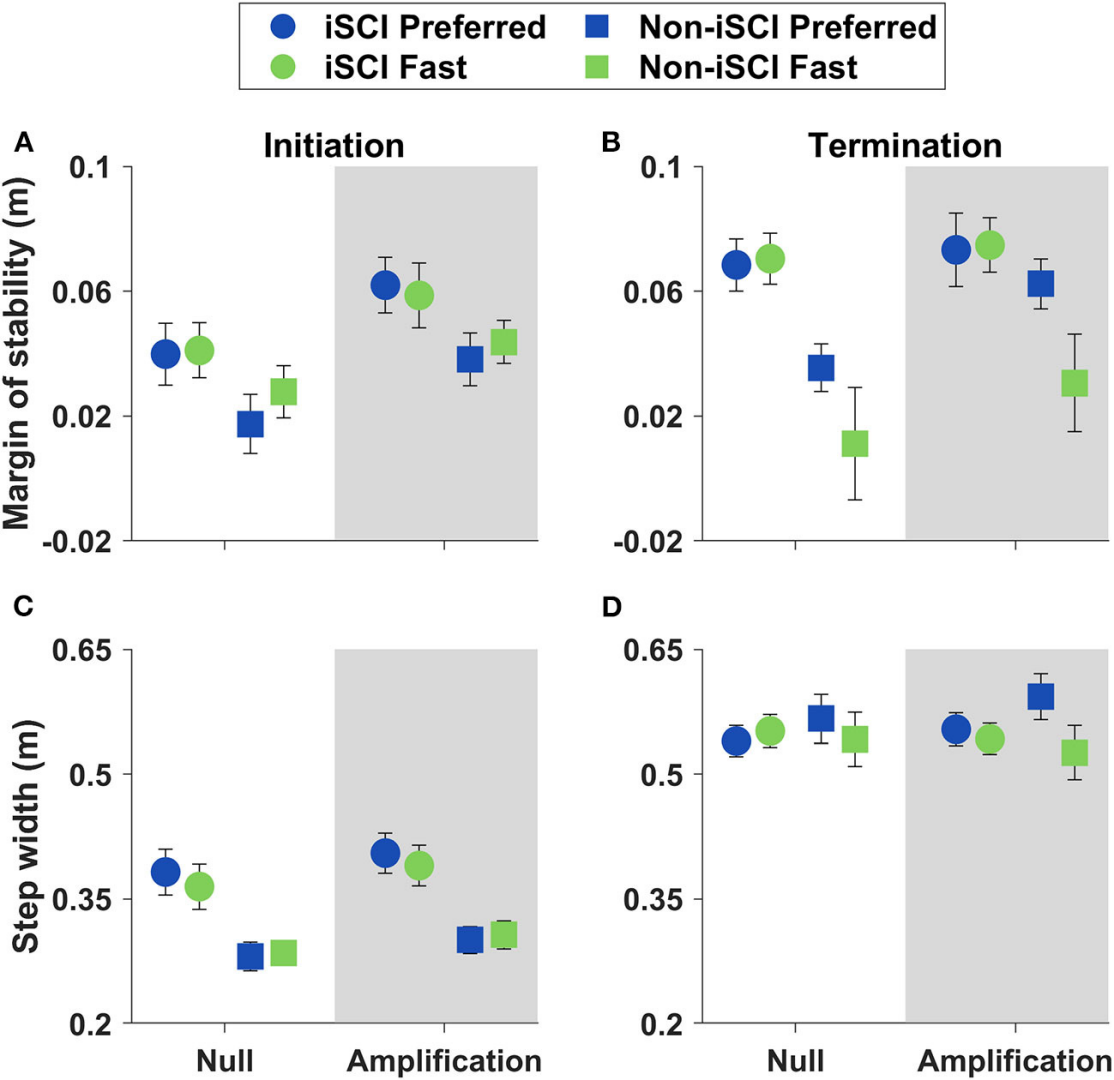
Means ± SE of MOS and step width on Initiation and Termination steps. **(A)** Initiation step MOS did not significantly change with speed within either field or group. **(B)** Termination step MOS was greater at the Preferred speed for the Non-iSCI group (*p* < 0.001) and within the Amplification field (*p* = 0.021). **(C)** Step width on the Initiation step did not significantly change with speed within either field or group. **(D)** Step width on the Termination step was greater at the Preferred speed for the Non-iSCI group (*p* = 0.002).

To assess maneuver strategies, we analyzed minimum lateral MOS and step width on the Initiation and Termination steps (Figure 5). Participants reduced their Initiation step MOS compared to both Baseline (*p* < 0.001) and Termination (*p* = 0.001) steps (Table 3). However, there was no effect of speed on the Initiation step MOS within either field (*p* > 0.350 for the two comparisons) or group (*p* > 0.200 for the two comparisons). In addition, the interaction between step, field, and speed did not significantly affect step width (*p* = 0.152), and there was no effect of speed on the Initiation step width within either group (*p* > 0.250 for the two comparisons). In summation, while the Initiation step MOS was significantly different from that during the other two steps evaluated, no interactions between speed and group or speed and field within the Initiation step were significant.

**Table 3:** MOS and step width model results comparing Baseline, Initiation, and Termination steps.

**Metric**	**Factor**	***P*-value**	**Sig. pairs**	***P*-value**
Minimum lateral margin of stability	Step	<0.001	**Initiation** **<** **Baseline**	**<0.001**
			**Initiation** **<** **Termination**	**0.001**
			Termination < Baseline	1.000
Step width	Step	<0.001	Initiation < Baseline	1.000
			**Initiation** **<** **Termination**	**<0.001**
			**Termination** **>** **Baseline**	**<0.001**

*Bold values highlight statistically significant results*.

On the Termination step, MOS was greater than that at the Initiation step (*p* = 0.001), and step width was greater compared to both the Initiation (*p* < 0.001) and Baseline (*p* < 0.001) steps (Table 3). The Termination step MOS decreased at the Fast speed within the Amplification field (*p* = 0.021) and within the Non-iSCI group (*p* < 0.001), while step width on the Termination step decreased at the Fast speed within the Non-iSCI group (*p* = 0.002) (Table 4). Overall, Termination step stability was significantly different from that at the other evaluated steps, as well as at the Fast speed in the Amplification field and the Non-iSCI group.

**Table 4:** MOS and step width model results comparing the interactions within the Termination step alone.

**Metric**	**Factor**	***P*-value**	**Sig. pairs**	***P*-value**
Lateral margin of stability	Step*Field*Speed	<0.001	**Amplification: Preferred** **>** **Fast**	**0.021**
			Null: Preferred > Fast	0.089
	Step*Group*Speed	<0.001	iSCI: Preferred < Fast	0.787
			**Non-iSCI: Preferred** **>** **Fast**	**<0.001**
Step width	Step*Field*Speed	0.152		
	Step*Group*Speed	<0.001	iSCI: Preferred < Fast	0.988
			**Non-iSCI: Preferred** **>** **Fast**	**0.002**

*Bold values highlight statistically significant results*.

#### Maneuver Performance Metric

**Figure 6:**
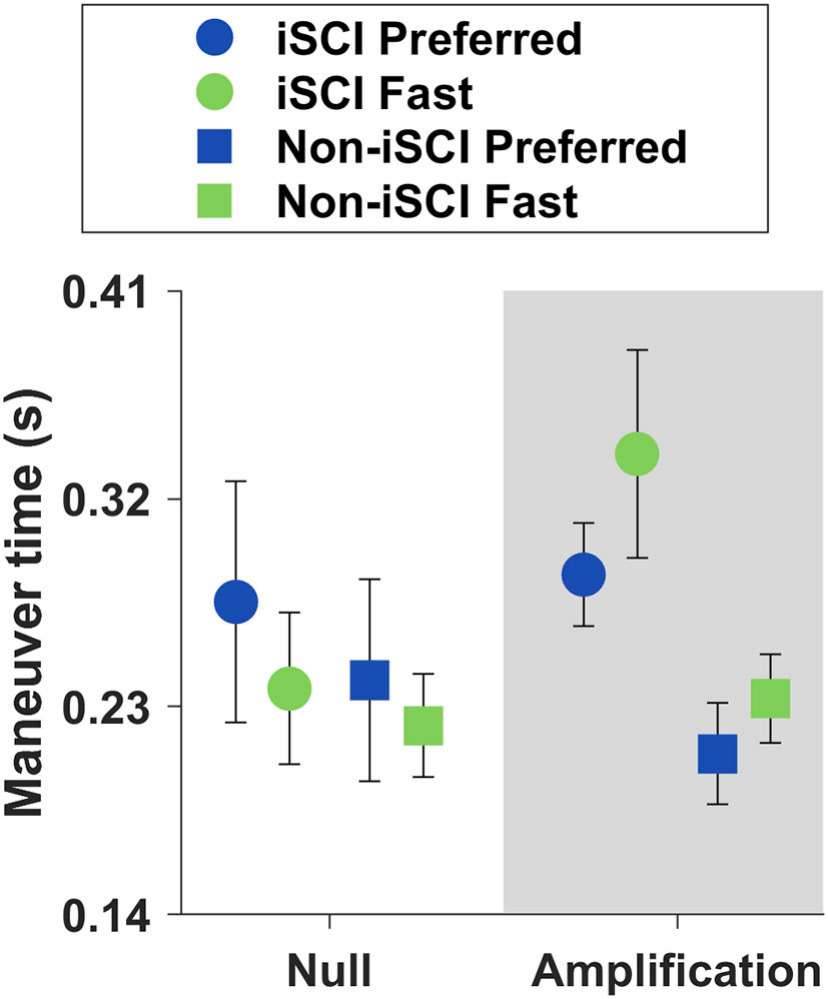
Means ± SE of maneuver times. There was no significant difference in maneuver time between Preferred and Fast speeds within the Null field (*p* = 1.000), but within the Amplification field, maneuvers were slower at the Fast speed than at the Preferred (*p* = 0.038).

To evaluate maneuver performance, we compared the time it took to complete the maneuvers across trials (Figure 6). A Friedman test yielded a significant difference between the maneuver times of the four trials (Chi-square = 11.450, *p* = 0.010). *Post-hoc* testing revealed that within the Amplification field, maneuvering took significantly longer when walking at the Fast speed compared to Preferred (*p* = 0.038). There was no significant difference in the maneuver time between the Preferred and Fast speeds in the Null field (*p* = 1.000).

## Discussion

The purpose of this study was to examine the effect of treadmill speed on gait stability (both straight walking and lateral maneuvers) in adults with and without iSCI. Walking trials at Preferred and Fast speeds were repeated in Null and Amplification force fields, with the latter field intended to probe stabilization strategies by challenging frontal plane COM control. During straight walking, individuals with and without iSCI showed no differences in lateral stability (Lyapunov exponent and MOS) between speeds, regardless of the type of the field. Increased step width variability at the Fast speed may have contributed to this preservation of COM stability as the correlation between lateral COM state and lateral foot placement strengthened with speed and in the Amplification field. To laterally maneuver, individuals decreased their minimum lateral MOS during the Initiation step and increased step width during the Termination step compared to straight walking. Speed did not affect Initiation step mechanics for either group, but individuals without iSCI decreased their Termination step MOS at the Fast speed, while those with iSCI maintained their Termination step stability across speeds, suggesting the use of a more cautious strategy.

### Straight Walking

Typically, straight walking performed on a treadmill poses few challenges to balance as it is performed with a constant speed and direction on a smooth, level surface free of obstacles. Therefore, we anticipated that the treadmill environment alone (Null field) would not provide an adequate challenge to observe any significant effects of forward walking speed on the control of lateral stability. In contrast, we hypothesized that in the Amplification field, individuals would exhibit a decrease in stability at the Fast speed compared to the Preferred.

#### COM Stability Did Not Change With Speed During Straight Walking

As anticipated, within the Null field, individuals with and without iSCI did not exhibit differences in lateral stability (Lyapunov exponent or MOS) between the Fast and Preferred walking speeds. These results support a previous study that also found no effect of speed on walking stability (14). However, it is possible that the effects of walking speed on lateral stability are small during treadmill use and more challenging environments are needed to detect them. Thus, we introduced the Amplification field with the hope that this balance-challenging environment would improve the sensitivity of lateral stability measures by accentuating even minor changes or errors. Compared to the Null field, Lyapunov exponent and MOS both increased in the Amplification field, indicating that the Amplification field was effective in challenging lateral stability, mirroring previous findings (16). However, this hypothesis that individuals would be less stable at the Fast speed compared to the Preferred when walking in the Amplification field was not supported. Despite the Amplification field creating the intended effect of challenging stability, walking speed did not affect Lyapunov exponent or MOS within either field. These results align with another study that found healthy individuals do not reduce their walking speed as a strategy to respond to lateral perturbations (40). Overall, the current findings suggest that individuals with and without iSCI can maintain lateral stability at different walking speeds, even when their lateral balance is challenged.

#### COM Excursion Changed With Speed, but Step Width Did Not

To understand why speed did not affect either measure of lateral stability, it is helpful to examine the two major contributing factors to these measures: COM dynamics and step width. At the Fast speed, peak lateral COM excursion decreased. This reduction in COM excursion is consistent with previous studies examining the effects of speed in younger adults (7, 41, 42), and may be expected because a faster cadence allows for less lateral movement before the next step. These results suggest that this relationship between speed and lateral COM excursion also holds for those with iSCI. While COM excursion decreased with speed, we did not find a significant change in step width between speeds. This observation is most consistent with studies in older adult populations (mean age in 70's) that found that at faster speeds, step width either follows a U-shaped curve (43) or does not significantly change (11, 44). For our sample populations (mean ages in the 50's), maintaining the step width at the Fast speed might have been a strategy to maintain lateral stability.

#### Steps Are More Variable and Coordinated With COM at Fast Speed

Step-to-step variability and its relationship with COM motion may encapsulate more of the nuanced requirements to control lateral stability when walking in a challenging environment. The current study found intra-subject step width variability to increase with speed, aligning with previous findings in younger populations (25, 42, 45). To better understand the meaning of step width variability in relation to stability, we also evaluated the ability of lateral COM state during midstance to predict the subsequent lateral foot placement (i.e., did greater COM motion to the right during the swing phase result in a right step positioned more laterally?). Along with more step width variability, we found a stronger correlation between COM state and lateral foot placement at the Fast speed and in the Amplification field. The current findings are consistent with the previous studies that also found a stronger COM-foot placement correlation in healthy individuals at faster speeds (25, 46) and in the Amplification field (16). Overall, these findings suggest that persons with and without iSCI similarly respond to faster gait speeds with greater step-to-step foot placement variability that is better coordinated with their COM state. If COM dynamics better predicted the next step placement in conditions that were potentially more challenging, this change in stepping may have helped participants to maintain lateral stability at the Fast speed and in the Amplification field.

We found the COM-foot placement coordination (Figure 4) to increase at the Fast speed, as well as in the Amplification field, indicating either changes to control based on the COM dynamics *or* a consistent error in these models. For example, step frequency also increased in both conditions, and perhaps timing falsely inflated the *R*^2^ values. A reduced stride time within these trials would allow stepping errors (i.e., foot placement further from that which is predicted by COM) less time to propagate. However, prior research refutes this potential explanation as other groups have found that with an increased step frequency, the corresponding correlation is weaker compared to normal walking (46, 47). Instead, the stronger correlation between COM state and subsequent foot placement may suggest important changes to the control mechanisms at the Fast speed and in the Amplification field.

Although the correlation between lateral COM state and foot placement changed with speed and field, we only see significant differences in lateral stability (Lyapunov exponent and MOS) across fields, not speeds. This discrepancy could be due to several reasons; for one, the Amplification field innately has a direct effect on mediolateral COM motion, while forward walking speed has greater effects on metrics in the sagittal plane, like step length and time. This suggests the challenge to lateral balance from speed alone was not as great as the challenge from the field. In addition, steps were wider in the Amplification field compared to Null but did not significantly change with speed, which may explain why MOS was greater in the Amplification field compared to the Null, but not at the Fast speed compared to Preferred. Therefore, the stronger correlation between lateral COM motion and foot placement suggests that stepping variance may be an important strategy to maintain a stable gait, rather than a direct measure of quantitative “stability.”

### Walking Maneuvers

As previously observed (19, 20, 48), we anticipated there would be differences in lateral MOS when comparing the Initiation and Termination steps of the maneuver to straight-ahead walking. A decrease in MOS during the Initiation step ipsilateral to the maneuver direction has been suggested to facilitate the maneuver by reducing the resistance of the body to lateral impulses (26, 48). With increases in walking speed, we expected further decreases in MOS during the Initiation step to enhance the capacity of the participant to rapidly maneuver. In contrast, during the Termination step, an increase in MOS can position the body to assist in breaking the lateral maneuver. Based on previous observations, we did not anticipate an effect of speed on the Termination step MOS during normal conditions (Null field) (19). Again, we introduced the Amplification field to further challenge lateral stability during the maneuvers to augment any effects of speed. We hypothesized that in the Amplification field, the lateral MOS on both the Initiation and Termination steps would be smaller during the Fast speed compared to the Preferred. If supported, these findings would suggest a potential speed-stability trade-off when initiating and arresting the lateral motion of maneuvers.

#### Maneuver Time Was Maintained or Slower at Fast Treadmill Speed

We anticipated that lateral maneuver speed would increase in proportion to increases in forward walking speed, but this did not occur. When walking in the Null field, we found no significant differences in the maneuver time between the Preferred and the Fast treadmill speeds. In the Amplification field, maneuvers were slower at the Fast treadmill speed. This important finding suggests that people with and without iSCI can independently regulate forward walking speed and lateral maneuver speed. Participants might have used a slower maneuvering strategy as a method to maintain lateral stability between the two treadmill speeds. While we only quantified four steps of the maneuver (two to initiate and two to terminate), participants could take additional steps between the Initiation and Termination steps to complete the maneuver. A non-parametric Friedman test found a significant difference in steps per trial (*p* = 0.048), but *post-hoc* comparisons with Bonferroni corrections were insignificant. On average, both groups took expectedly fewer steps to maneuver in the Null field at the Fast speed compared to the Preferred, but in the Amplification field, both groups took more steps at the Fast treadmill speed. Participants without iSCI took 4.16 and 4.22 steps and those with iSCI took 4.27 and 4.45 steps to complete a maneuver in the Amplification field at the Preferred and Fast treadmill speeds, respectively. Taking more time to complete the lateral maneuver and distributing the maneuver over more steps should improve the lateral stability by reducing peak lateral velocities and improving the capacity to maintain the lateral COM position within the BOS. These findings are in line with a prior study suggesting that older adults will use multiple steps to execute maneuvers as a method to maintain stability (49). Independent control of lateral maneuver speed may be an important strategy used by individuals with iSCI to maintain lateral stability when moving at faster forward walking speeds.

#### Initiation Step MOS Did Not Change With Speed

To initiate the maneuver, participants reduced their lateral MOS in comparison to straight-walking steps, as has been found in previous studies (19, 20, 26). Decreasing the lateral MOS is beneficial when initiating a volitional maneuver as a smaller impulse is then necessary to move the COM in the maneuver direction. Interestingly, there was no effect of forward walking speed on the Initiation step MOS or step width. While we expected the Amplification field to magnify the effects of speed on the Initiation step MOS, our findings did not support this, which is also likely due to the slower maneuvers performed at the Fast treadmill speed. Overall, treadmill speed did not significantly affect Initiation step stability in either field or group, suggesting that forward walking speed alone may not change the lateral maneuver initiation strategy.

The absence of a smaller Initiation step MOS at the Fast speed in the Null field seems to contrast with a previous study. Viramontes et al. had participants with iSCI perform single lateral “lane-change” maneuvers during overground walking and found the Initiation step MOS to decrease with increasing maneuver speed (19). In this prior experiment, the maneuvers were successfully performed at different speeds. As discussed earlier, the current experiment did not result in significant differences in maneuver time between the Fast and Preferred treadmill speeds. Our expectation that individuals would reduce MOS at the Fast treadmill speed assumed that this change would be beneficial for rapidly initiating a lateral maneuver. However, these findings that participants selected strategies that maintained or decreased lateral maneuver speeds best explains why we did not observe further reductions in Initiation step MOS in the current study.

#### Termination Step MOS Reduced at “Fast” Speed in the Amplification Field

Participants arrested the maneuvers by maintaining a lateral MOS on the Termination step that was not significantly different from straight walking. To produce this lateral MOS, we observed a greater step width during the Termination step compared to both the Initiation step and straight walking. This strategy to arrest lateral momentum and prevent target overshoot during maneuvers has been previously observed in iSCI populations (19, 20).

Based on prior observations (19), we anticipated that speed would not have a significant effect on Termination step stability in the Null field. In contrast, we expected the Amplification field to increase the challenge to arrest lateral motion, so we hypothesized there would be a reduction in the Termination step MOS at the Fast treadmill speed compared to the Preferred. This hypothesis was supported. However, it is important to note that this decrease in the Termination step MOS at the Fast treadmill speed in the Amplification field was predominantly driven by the group without iSCI. Data in Figure 5 illustrate that individuals with iSCI maintained their Termination step MOS between speeds, while individuals without iSCI decreased the Termination step MOS at the Fast speed compared to the Preferred.

Individuals with and without iSCI utilized different approaches to maneuvering in the Amplification field at the Fast treadmill speed. Individuals with iSCI took more steps and time, a strategy that allowed them to maintain their Termination step MOS between the speed conditions. While individuals without iSCI also took more steps and time at the Fast treadmill speeds, these changes were not as pronounced as within the iSCI group. As a result, individuals without iSCI exhibited a reduction in their Termination step MOS at the Fast speed. The varying maneuver strategies potentially highlight the differing priorities between the groups. The participants with iSCI, a population more susceptible to falling, likely prioritized lateral stability. This group reduced the rate at which they performed the maneuver, which allowed them to maintain a consistent safety level across the two treadmill speeds. This strategy by the group with iSCI to prioritize stability only became evident in the Amplification field that increased the challenges to control lateral velocity. In contrast, the group without iSCI chose maneuver strategies that may have prioritized maneuver performance and resulted in reductions in lateral MOS during the Termination step at the Fast treadmill speed. Prior research has found stability-maneuverability trade-offs during lateral walking maneuvers (48). The current research builds on this idea by demonstrating that in balance-challenging situations, individuals with iSCI employ maneuver strategies to maintain stability.

### Conclusion

These findings suggest that ambulatory individuals with iSCI modify their gait patterns to maintain stability as walking speed increases. These strategies allowed them to successfully maintain lateral stability during straight walking at faster speeds, even in balance-challenging environments. However, at the Fast speed, both groups reduced lateral COM excursion and increased step-to-step foot placement variability. These kinematic changes may have been beneficial for controlling lateral COM motion as the relationship between lateral COM state and foot placement was stronger at the Fast speed. We also found that both groups were able to successfully perform lateral maneuvers at fast treadmill speeds. Notable differences in stabilization strategies between the groups emerged when terminating maneuvers in the Amplification field. Individuals without iSCI reduced MOS upon maneuver termination at the Fast speed, while those with iSCI appear to have prioritized stability over speed by choosing maneuver strategies (e.g., taking more steps and time) that maintained their lateral MOS across treadmill speeds.

While our participants with iSCI were considered high functioning, half reported falling one or more times in the past year, which demonstrates that stability remains an issue. In this study, they successfully maintained gait stability during fast straight walking, but these findings suggest that doing so during fast maneuvers may be a challenge. Real-world situations arise when a person is required to negotiate obstacles with both speed and stability, such as avoiding traffic when crossing a street. For high-functioning individuals with iSCI, physical therapy interventions that concurrently challenge speed and lateral stability may be needed to help them learn to accomplish such complex tasks that are common to community ambulation.

## Data Availability Statement

The original contributions presented in the study are included in the article/Supplementary Material. Further inquiries can be directed to the corresponding author/s.

## Ethics Statement

The studies involving human participants were reviewed and approved by Edward Hines Jr. VA Hospital and Northwestern University Institutional Review Boards. The patients/participants provided their written informed consent to participate in this study. Written informed consent was obtained from the individual(s) for the publication of any potentially identifiable images or data included in this article.

## Author Contributions

TC, WO, and KG collaborated on the study concept and design. TC, JW, WO, and KG assisted in data collection. TC performed data and statistical analysis. TC and KG drafted the manuscript. All authors contributed to the revisions and in the approval of the submitted version.

## Conflict of Interest

The authors declare that the research was conducted in the absence of any commercial or financial relationships that could be construed as a potential conflict of interest.

## Publisher's Note

All claims expressed in this article are solely those of the authors and do not necessarily represent those of their affiliated organizations, or those of the publisher, the editors and the reviewers. Any product that may be evaluated in this article, or claim that may be made by its manufacturer, is not guaranteed or endorsed by the publisher.

## Supplementary Material

Supplementary Video 1 A representative participant with iSCI first performing straight walking in the target lane and then transitioning to a series of lateral maneuvers.

Supplementary Table 1 Complete dataset for straight-walking and maneuver metrics.
